# Identification of intestinal flora-related key genes and therapeutic drugs in colorectal cancer

**DOI:** 10.1186/s12920-020-00810-0

**Published:** 2020-11-16

**Authors:** Jiayu Zhang, Huaiyu Zhang, Faping Li, Zheyu Song, Yezhou Li, Tiancheng Zhao

**Affiliations:** 1 Department of Gastrointestinal Colorectal and Anal Surgery, China-Japan Union Hospital of Jilin University, Changchun, Jilin China; 2 Department of Urology, The First Hospital of Jilin University, Changchun, Jilin China; 3 Department of Vascular Surgery, China-Japan Union Hospital of Jilin University, Changchun, Jilin China; 4 Department of Endoscopy Center, China-Japan Union Hospital of Jilin University, Changchun, Jilin China

**Keywords:** Colorectal cancer, Intestinal flora, Text mining, Key genes, Drugs

## Abstract

**Background:**

Colorectal cancer (CRC) is a multifactorial tumor and a leading cause of cancer-specific deaths worldwide. Recent research has shown that the alteration of intestinal flora contributes to the development of CRC. However, the molecular mechanism by which intestinal flora influences the pathogenesis of CRC remains unclear. This study aims to explore the key genes underlying the effect of intestinal flora on CRC and therapeutic drugs for CRC.

**Methods:**

Intestinal flora-related genes were determined using text mining. Based on The Cancer Genome Atlas database, differentially expressed genes (DEGs) between CRC and normal samples were identified with the limma package of the R software. Then, the intersection of the two gene sets was selected for enrichment analyses using the tool Database for Annotation, Visualization and Integrated Discovery. Protein interaction network analysis was performed for identifying the key genes using STRING and Cytoscape. The correlation of the key genes with overall survival of CRC patients was analyzed. Finally, the key genes were queried against the Drug-Gene Interaction database to find drug candidates for treating CRC.

**Results:**

518 genes associated with intestinal flora were determined by text mining. Based on The Cancer Genome Atlas database, we identified 48 DEGs associated with intestinal flora, including 25 up-regulated and 23 down-regulated DEGs in CRC. The enrichment analyses indicated that the selected genes were mainly involved in cell–cell signaling, immune response, cytokine-cytokine receptor interaction, and JAK-STAT signaling pathway. The protein–protein interaction network was constructed with 13 nodes and 35 edges. Moreover, 8 genes in the significant cluster were considered as the key genes and chemokine (C-X-C motif) ligand 8 (CXCL8) correlated positively with the overall survival of CRC patients. Finally, a total of 24 drugs were predicted as possible drugs for CRC treatment using the Drug-Gene Interaction database.

**Conclusions:**

These findings of this study may provide new insights into CRC pathogenesis and treatments. The prediction of drug-gene interaction is of great practical significance for exploring new drugs or novel targets for existing drugs.

## Background

Colorectal cancer (CRC) has the third-highest incidence rate and the second-highest mortality rate among all types of cancers worldwide, according to the 2018 global cancer statistics [1]. The World Health Organization estimates that the prevalence of CRC will rise to 3093 million by 2040 [2]. Although great effort has been made to improve clinical treatment over the past few decades, CRC still poses an enormous threat to human health. For example, the median overall survival rarely exceeds 6.8 months for patients with metastatic CRC [3, 4], and the 5-year survival rate remains only 10% [5]. Therefore, targeting novel pathways is indispensable for further improvement in the prognosis of patients with CRC.

CRC is generally considered to be a multifactorial disease involving diet, inflammatory process, genetic alteration, and environmental factor [6, 7]. The human intestinal tract contains 10 to 100 trillion florae, which is 10 times more than the number of total human body cells. There is increasing evidence that patients with CRC harbor a distinct microbiota. Compared with healthy individuals, the probiotics, including *Bifidobacterium* and *Lactobacillus acidophilus,* are decreased while the pathogenic bacteria, including *Bacteroides/Prevotella* and *Enterococcus faecalis,* are increased in patients with CRC [8–11]. Previous studies have confirmed the role of intestinal flora as an environmental factor for the carcinogenesis of CRC [12, 13]. Intestinal flora dysfunction can induce abnormal immune reactions, resulting in a special immune microenvironment in colorectal tissue and producing carcinogenic metabolites that induce DNA damage and gene mutations in host cells [14]. For example, alterations in the microbiota can drive the upregulation of interleukin-17c in intestinal epithelial cells during intestinal tumorigenesis. Microbiota-driven IL-17c promotes cell survival and tumorigenesis by inducing the expression of Bcl-2 and Bcl-xl in intestinal epithelial cells [15]. The enzymes produced by *Clostridium* bacteria induce increased secondary bile acids that lead to ROS production and DNA damage. These changes can lead to non-integer multiples, KRAS mutations, and micronuclei [16]. However, the current knowledge on the mechanism by which intestinal flora influences the pathogenesis of CRC remains scarce.

Due to the existence of tens of thousands of biomedical journals, the field of biomedicine is flooded with new articles, leading to information overload. Therefore, there is increasing interest in text mining, an efficient approach that enables the identification of biologically relevant entities, such as genes and diseases, complex biological relations, and comprehensive networks. Researches in multifarious fields have been performed using text mining, ranging from pattern-matching methods to molecular events extraction [17–19]. Yet to date, the approaches of text mining have not addressed how intestinal flora has an impact on the development of CRC.

In this study, we extracted the genes associated with intestinal flora using text mining. Then, we generated common genes by combining these extracted genes and differentially expressed genes (DEGs) between CRC and normal samples. With further analyses of the functional enrichment and protein–protein interaction (PPI), we identified 8 potential target genes. Finally, candidate drugs for CRC treatment were derived using the Drug-Gene Interaction database (DGIdb).

## Methods

### Gene collection

The web server GenCLiP 3 (https://ci.smu.edu.cn/genclip3/analysis.php) was used to perform text mining. The inclusion criteria for screening were: (i) literature published from 1980 to 2020; (ii) human genes that co-occur with “gut microbiota” or “intestinal flora” in sentence (iii) genes in MEDLINE. Subsequently, a list of genes associated with intestinal flora was extracted. To identify the DEGs between CRC and normal samples from The Cancer Genome Atlas (TCGA) database, the matrix data of gene expression levels were analyzed with the limma package of the R software. Log_2_ (fold change) > 2 or < − 2 and false discovery rate < 0.05 were set as the threshold for filtering DEGs. VennDiagram package was used to identify the intersection of the two aforementioned gene sets.

### Functional enrichment analyses

Using the online tool Database for Annotation, Visualization and Integrated Discovery (DAVID), enrichment analyses of Gene Ontology (GO) and Kyoto Encyclopedia of Genes and Genomes (KEGG) pathway were performed for the intersection genes. *P* value < 0.05 was considered as statistically significant.

### PPI network analysis

The PPI network of the selected common genes was constructed using the online STRING (https://string-db.org/) and then was visualized using Cytoscape v3.7.2. A confidence score of 0.4 or higher was set as the cut-off criterion. The Molecular Complex Detection (MCODE) plug-in of Cytoscape was used to classify the significant node clusters with the default. The genes in the most significant cluster were selected as key genes for overall survival and drug-gene interaction analyses.

### Correlation of the key genes with overall survival

Gene Expression Profiling Interactive Analysis (GEPIA) is a web-based tool that is freely available to users [20]. Based on TCGA data, GEPIA contains multiple modules, including analyses of the relationship between gene expression and overall survival. We used the GEPIA to analyze the correlation of the key genes with overall survival of CRC patients.

### Drug-gene interaction

The screened key genes were served as the potential targets for searching drugs. DGIdb database (v3.0, https://www.dgidb.org/), a freely available database, was used to identify known and potential drug-gene interactions. Finally, the FDA approved antineoplastic drugs were considered as the potential drugs for CRC treatment.

## Results

### Identification of intestinal flora-related DEGs in CRC

**Fig. 1:**
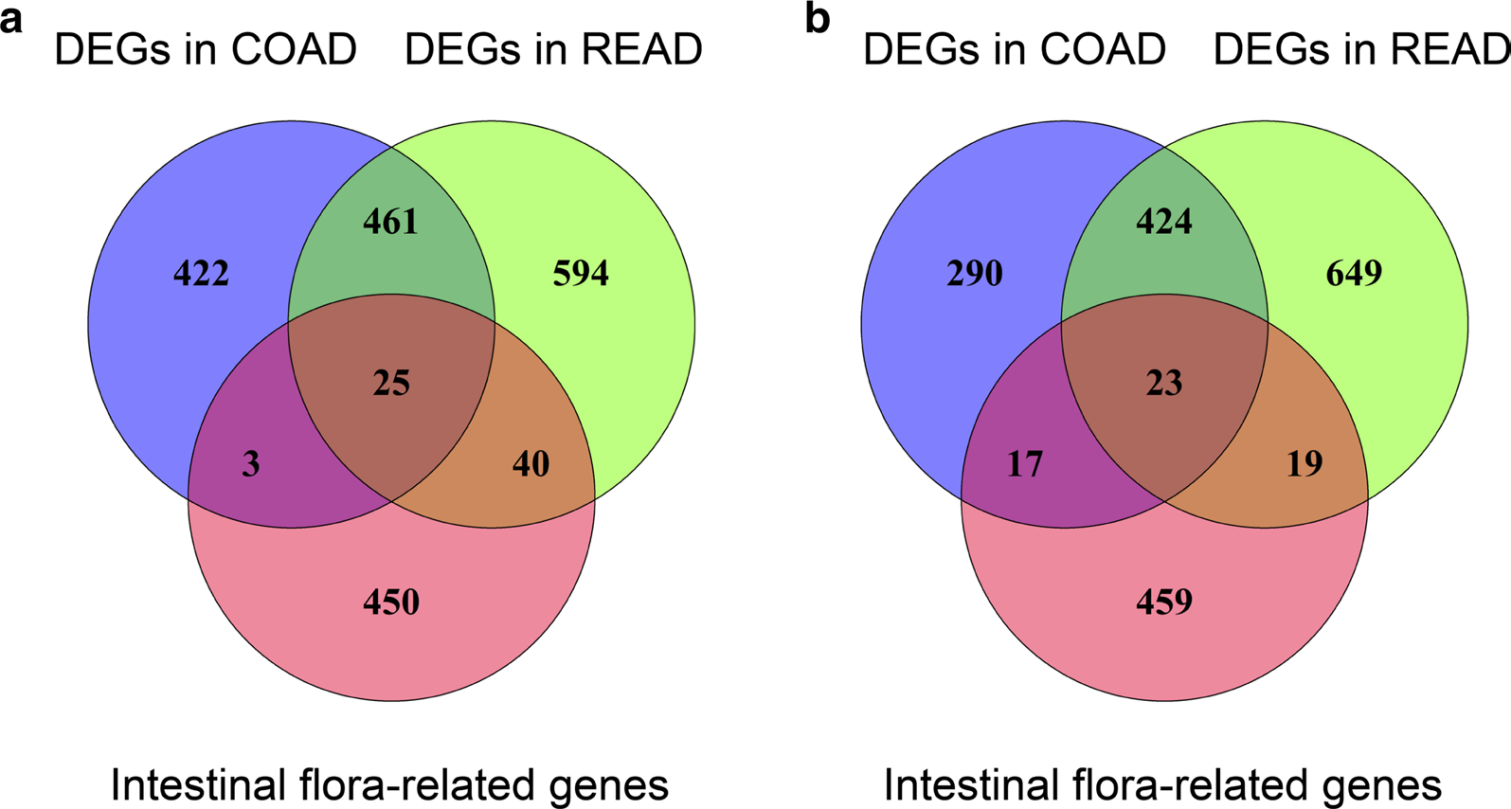
Intersection between intestinal flora-related genes and DEGs in CRC. **a** Venn diagrams showing the overlapping up-regulated genes among intestinal flora-related genes, DEGs in COAD, and DEGs in READ. **b** Venn diagrams showing the overlapping down-regulated genes among intestinal flora-related genes, DEGs in COAD, and DEGs in READ. CRC, colorectal cancer; DEGs, differentially expressed genes; COAD, colon adenocarcinoma; READ, rectal adenocarcinoma

According to the inclusion criteria, 518 genes were identified within 1110 published papers. To explore whether intestinal flora influences the pathogenesis of CRC by regulating the gene expression, we then identified the DEGs between CRC and normal samples from the TCGA database. Compared with the normal samples, there were 1665 and 2235 DEGs in colon adenocarcinoma (COAD) and rectal adenocarcinoma (READ), respectively. Moreover, there were 25 up-regulated genes (Fig. 1a) and 23 down-regulated genes (Fig. 1b) in common between the DEGs and intestinal flora-related genes. The 48 common genes listed in Table 1 were selected for further analysis.

**Table 1:** Genes in common between the DEGs and intestinal flora-related genes

Category	Gene symbol
Up-regulated	CDKN2A, TNFSF9, CXCL1, CSF2, SPP1, MMP3, MUC5AC, FUT1, NPC1L1, CLDN2, NOX4, GAST, GATA4, IGF2, IL23A, ALB, CXCL8, CXCL10, OSM, REG1A, HP, FGF19, IL22, DIO2, AGT
Down-regulated	DAO, PYY, ALPI, NPY, SST, ABCG2, FABP2, IL6R, CASR, CNR1, GPR15, CHGA, SLCO4C1, DNASE1L3, AQP8, MAOB, GPT, INSL5, MAPT, PRKCB, GCG, CD36, CFD

DEGs, differentially expressed genes

### Functional enrichment analyses

**Fig. 2:**
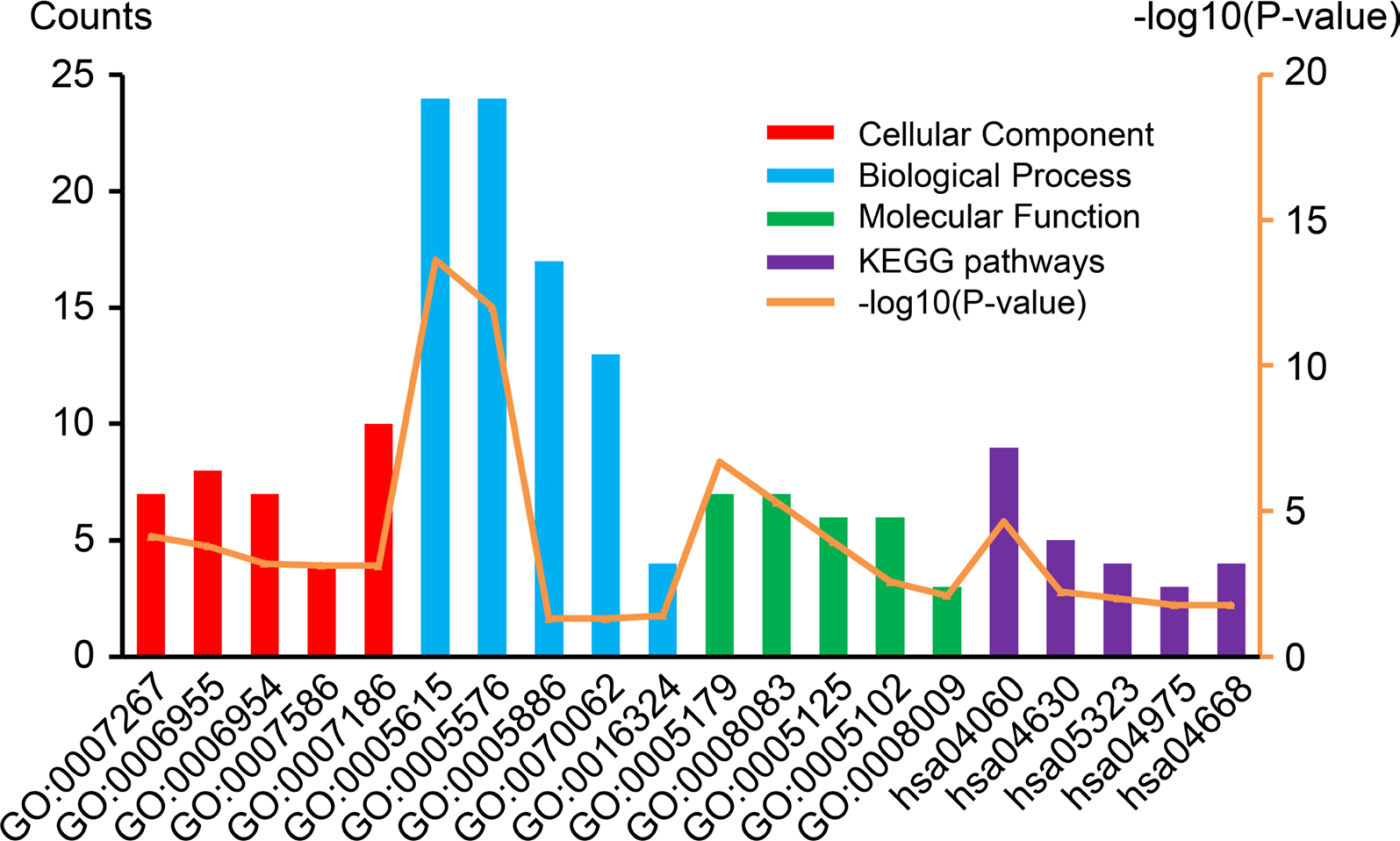
Functional enrichment analyses of the 48 intestinal flora-related DEGs**.** The horizontal axis denotes different KEGG pathways and GO terms, including molecular function (MF), cellular component (CC), and biological process (BP). The vertical axis denotes the number of enriched genes and significant differences. DEGs, differentially expressed genes; GO, gene ontology; KEGG, Kyoto Gene and Genomic Encyclopedia

Using the DAVID tool, we identified 55 GO terms and 5 KEGG terms in which the 48 intestinal flora-related DEGs enriched significantly (*P* < 0.05). The results showed the top 5 significant enrichment terms for biological processes, cellular component and molecular function, and 5 KEGG pathway terms (Fig. 2). As listed in Table 2, in the biological processes annotation, selected DEGs are mainly involved in cell–cell signaling, immune response, inflammatory response, digestion, and G-protein coupled receptor signaling pathway. In the cellular component annotation, DEGs are mainly involved in extracellular space, extracellular region, plasma membrane, extracellular exosome, and apical plasma membrane. As for molecular function, DEGs are mainly involved in hormone activity, growth factor activity, cytokine activity, receptor binding, and chemokine activity.

**Table 2:** The top five GO enrichment terms of the intestinal flora-related DEGs

Category	Term	Description	Count	*P* value
BP	GO:0007267	cell–cell signaling	7	7.21E−05
BP	GO:0006955	immune response	8	1.57E−04
BP	GO:0006954	inflammatory response	7	6.24E−04
BP	GO:0007586	digestion	4	7.25E−04
BP	GO:0007186	G-protein coupled receptor signaling pathway	10	7.52E−04
CC	GO:0005615	extracellular space	24	2.29E−14
CC	GO:0005576	extracellular region	24	9.97E−13
CC	GO:0005886	plasma membrane	17	4.90E−02
CC	GO:0070062	extracellular exosome	13	4.93E−02
CC	GO:0016324	apical plasma membrane	4	3.90E−02
MF	GO:0005179	hormone activity	7	1.99E−07
MF	GO:0008083	growth factor activity	7	4.85E−06
MF	GO:0005125	cytokine activity	6	1.13E−04
MF	GO:0005102	receptor binding	6	2.64E−03
MF	GO:0008009	chemokine activity	3	7.88E−03

*GO* gene ontology, *DEGs* differentially expressed genes, *BP* biological process, *CC* cellular components, *MF* molecular function

Of the 5 enriched pathways, the two most significant pathways were cytokine-cytokine receptor interaction (*P* = 2.22E−05) and JAK-STAT signaling pathway (*P* = 0.006). Additional highly enriched relevant pathways were rheumatoid arthritis, fat digestion and absorption, and TNF signaling pathway (Table 3).

**Table 3:** The KEGG pathway enrichment terms of the intestinal flora-related DEGs

Term	Description	Count	*P* value
hsa04060	Cytokine-cytokine receptor interaction	9	2.22E−05
hsa04630	JAK-STAT signaling pathway	5	5.97E−03
hsa05323	Rheumatoid arthritis	4	9.85E−03
hsa04975	Fat digestion and absorption	3	1.66E−02
hsa04668	TNF signaling pathway	4	1.67E−02

*KEGG* Kyoto Encyclopedia of Genes and Genomes, *DEGs* differentially expressed genes, *JAK* Janus kinase, *STAT* signal transducer and activator of transcription, *TNF* tumor necrosis factor

### PPI network construction and survival analysis

**Fig. 3:**
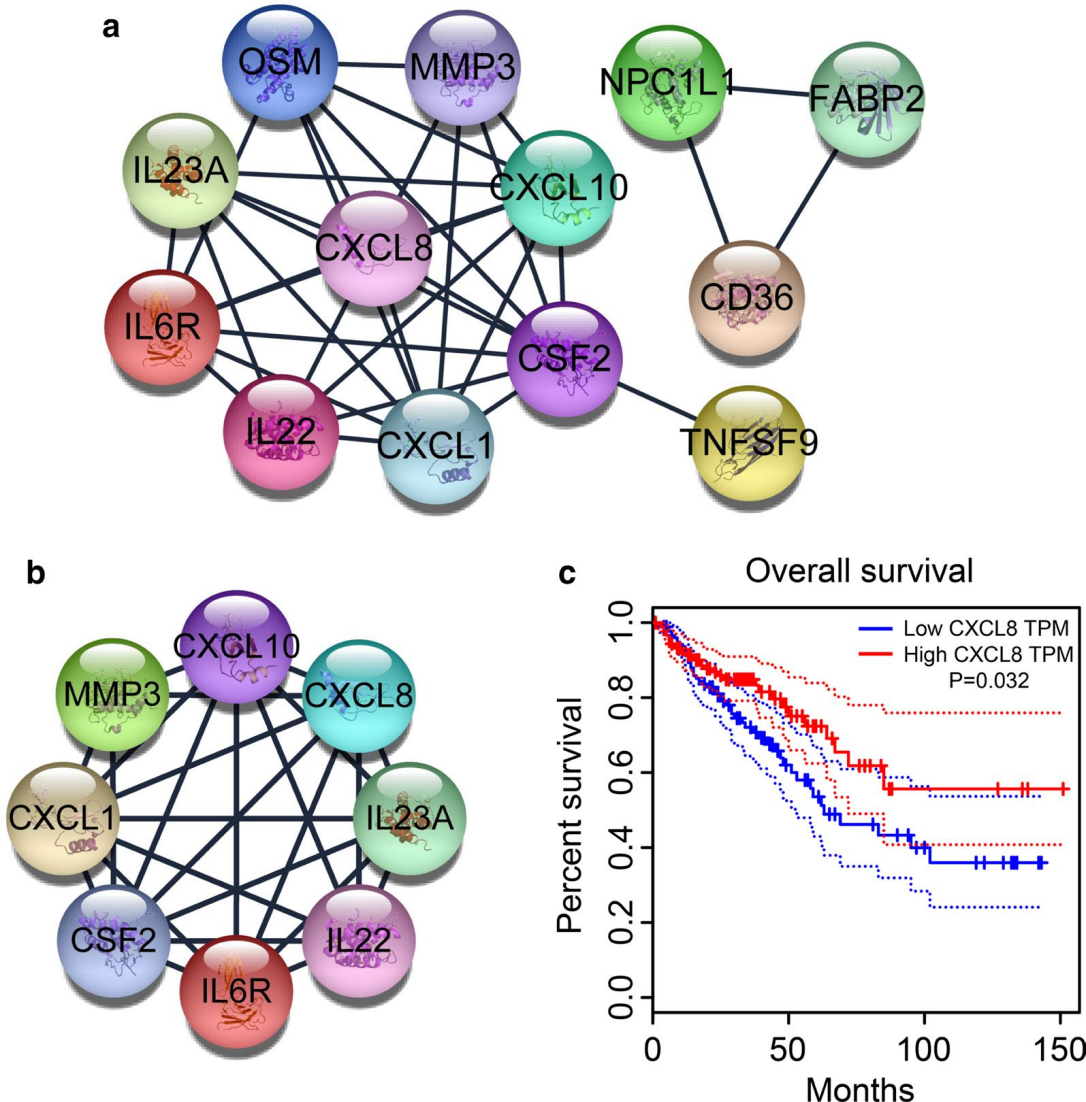
PPI network and survival analysis. **a** Based on the STRING online database, 13 intestinal flora-related DEGs were filtered into PPI network. **b** The most significant module from the PPI network. **c** Kaplan–Meier curves of overall survival for patients grouped by expression level of CXCL8. PPI, protein–protein interaction; DEGs, differentially expressed genes; CXCL8, chemokine (C-X-C motif) ligand 8

From the KEGG pathway analysis, 13 genes were selected. With these 13 genes, the PPI network analysis was performed using STRING and then was visualized using Cytoscape v3.7.2 (Fig. 3a). There were 13 nodes and 35 edges in the PPI network (PPI enrichment *P* < 1.0E−16). Moreover, the most significant cluster network (score = 7.143) was created using MCODE plug-in, consisting of 25 edges and 8 nodes/genes (CSF2, CXCL1, MMP3, CXCL10, IL23A, CXCL8, IL22, and IL6R) (Fig. 3b).

Further, we used the GEPIA to analyze the correlation of the 8 key genes with overall survival of CRC patients. The result showed that only chemokine (C-X-C motif) ligand 8 (CXCL8) was closely related to the overall survival of patients with CRC (Fig. 3c).

### Drug‑gene interaction

The 8 potential key genes in the PPI network were selected for drug-gene interaction analysis. A list of 24 drugs meeting the requirements for CRC treatment was compiled, comprising only the antineoplastic drugs that had been approved by the FDA (Table 4). As listed in Table 4, the potential targets of these drugs include CSF2, CXCL8, IL6R, and CXCL10, and 79.2% (19/24) of the drugs target CSF2 and CXCL8. Several drugs have been applied either alone or in combination in clinical trials or treatments for CRC, such as cetuximab, oxaliplatin, bevacizumab, temozolomide, and interferon alfa-2b.

**Table 4:** Candidate drugs targeting key genes

Gene	Drug	Score^*^	Refs. (PMID)
CSF2	Interferon alfa-2b	2	10522033
CSF2	Mechlorethamine	2	10640980
CSF2	Streptozotocin	2	16342200
CSF2	Procarbazine	2	10640980
CSF2	Temozolomide	3	12610499, 16100942
CSF2	Mycophenolic acid	2	9822358
CSF2	Nordihydroguaiaretic acid	2	2453577
CSF2	Idarubicin	2	8915668
CSF2	Cytarabine	3	8819077, 8450676
CSF2	Vinblastine	2	10640980
CXCL8	Paclitaxel	2	9271387
CXCL8	Bevacizumab	1	–
CXCL8	Aspirin	2	12576442
CXCL8	Leflunomide	2	10902750
CXCL8	Tretinoin	2	8900181
CXCL8	Cetuximab	4	10614716, 15908664, 10037173
CXCL8	Medroxyprogesterone acetate	2	15914533
CXCL8	Cyclophosphamide	1	–
CXCL8	Verapamil	2	2686646
IL6R	Oprelvekin	1	–
IL6R	Tocilizumab	8	16899109
IL6R	Fluorouracil	2	8888499
IL6R	Thalidomide	2	12515619
CXCL10	Oxaliplatin	2	16101140

^*^The score is the combined number of database sources and PubMed references supporting a given interaction

## Discussion

The intestinal flora plays significant roles in the formation and development of CRC via producing carcinogenic toxins and metabolites, causing intestinal dysbiosis, and altering immune response [14]. However, current knowledge of the specific mechanism by which intestinal flora influences the pathogenesis of CRC remains limited. In this study, we utilized text mining to extract the intestinal flora-related genes. With further analyses of the functional enrichments and PPI network, we identified 8 key genes associated with intestinal flora and the development of CRC. Finally, candidate drugs targeting 4 key genes (CSF2, CXCL8, IL6R, and CXCL10) were derived using the DGIdb database.

To explore whether intestinal flora contributes to the development of CRC via regulating gene expression, we identified the DEGs between CRC and normal samples. The interaction of the DEGs and intestinal flora-related genes extracted by text mining was selected for further functional enrichments and PPI analyses. Functional enrichment of the 48 selected DEGs in the GO biological process and KEGG pathways highlight their role in immune response, inflammatory response, and intestinal function. Previous studies have confirmed that alteration of intestinal flora can promote CRC development by inducing inflammation, immune suppression, and attacking the gut barrier system [21–23]. Moreover, intestinal flora has been proven to promote tumorigenesis and chemoresistance of CRC via regulating gene expression [24–26]. Therefore, the mechanism of intestinal flora contributing to the development of CRC is complex and multifaceted.

Using the MCODE plug-in, we created the most significant cluster network, including 8 genes. 87.5% (7/8) of these genes enriched in the cytokine-cytokine receptor interaction pathway, indicating that interactions between the intestinal flora and immune system contribute to the development of CRC.

Drug resistance has been a Gordian knot in the treatment of cancer. Therefore, we identified a list of 24 drugs with the potential therapeutic efficacy against CRC. Among the 8 key genes, the potential gene targets of these drugs are CSF2, CXCL8, IL6R, and CXCL10, and most of the drugs were CSF2 and CXCL8 inhibitors. As a cytokine, CSF2 can stimulate the recruitment and maturation of dendritic cells to induce protective immunity and then exert anti-tumor effects [27]. Yet in the tumor microenvironment, CSF2 is often up-regulated and suppress the immune response, resulting in a poor prognosis for patients [27, 28]. Another cytokine, CXCL8, has been proven to be associated with chemoresistance of CRC [29]. The increased expression of CXCL8 induced by anti-cancer drugs, such as doxorubicin and cisplatin, can upregulate the expression of ATP-binding cassette transporters, resulting in poor chemotherapeutic response [29]. Moreover, a high level of CXCL8 predicted poor overall survival in patients with CRC [30], which is contrary to our findings in this study. This discrepancy probably attributes to the expression characteristics of CXCL8 in different cells within CRC tissues. The tumor microenvironment is a complex system that contains infiltrating immune cells, epithelial cells, and fibroblasts, as well as tumor cells. In addition, CXCL8 is a pro-inflammatory cytokine produced by tumor cells, neutrophils, and endothelial cells [31]. Oladipo et al. found that CRC patients with CXCL8 positivity in the tumor-infiltrating cells had a significantly improved prognosis compared with patients with negativity [32]. Therefore, we speculated that CXCL8 expression in infiltrating cells was dominant in CRC tissues that were obtained from the TCGA database. Among the listed drugs, cetuximab was considered as a prospective drug for CRC therapy thanks to its ability to decrease CXCL8 expression other than inhibiting the epidermal growth factor receptor [33, 34].

IL6R is the receptor of IL-6, which forms a dimer with glycoprotein-130. IL-6 binds the IL6R to initiate the IL-6 signaling that transduces intracellular signals via activation of the JAK-STAT3 pathway [35]. Research has suggested that the IL-6 signaling pathway plays an important role in the development and chemoresistance of various cancers, including CRC [36–38]. Accordingly, IL6R has been proposed as a promising target for CRC treatment. IL6R antagonist antibody, tocilizumab, could significantly reduce viability and enhance the apoptosis of CRC cells by blocking the IL-6/STAT3 pathway [39].

CXCL10 is a member of interferon-inducible proteins, which is increasingly being considered as a pro-tumorigenic factor in various cancers, including CRC [40]. Besides, elevated serum CXCL10 was associated with liver metastasis and poor prognosis in CRC [41]. Thus, CXCL10 may be a potential therapeutic target for CRC.

## Conclusions

In conclusion, we presented a novel method to explore the molecular mechanism underlying the effect of intestinal flora on CRC. Importantly, we identified 8 potential key genes and 24 candidate drugs. Ten of the 24 drugs have not been tested in CRC, which not only provides a theoretical basis for new trials but also provides new insights into targeting drug discovery. However, our study has some limitations. First, a limitation of the present study is its retrospective nature. All the findings were generated based on the published literature and public database. More prospective studies should be required to verify our findings. Second, it was not clear whether the decrease in probiotics or the increase in pathogenic bacteria caused the DEGs in CRC. Therefore, further mechanistic study of intestinal flora-related genes is encouraged.

## Data Availability

A list of genes associated with intestinal flora was extracted using the publicly available GenCLiP3 database (https://ci.smu.edu.cn/genclip3/analysis.php). The microarray studies of CRC analyzed during the current study were available in The Cancer Genome Atlas (TCGA) (dataset ID: TCGA-COAD, https://gdc.xenahubs.net/download/TCGA-COAD.htseq_counts.tsv.gz; dataset ID: TCGA-READ, https://gdc.xenahubs.net/download/TCGA-READ.htseq_counts.tsv.gz). The online tool DAVID (https://david.ncifcrf.gov/) was used to perform functional enrichment analyses. The PPI network of the selected common genes was constructed using the online STRING (https://string-db.org/). Gene Expression Profiling Interactive Analysis (GEPIA) tool (https://gepia.cancer-pku.cn/index.html) to analyze the correlation of the key genes with overall survival of CRC patients. DGIdb database (https://www.dgidb.org/) was used to identify known and potential drug-gene interactions.

## Abbreviations

CRC: Colorectal cancer
DEGs: Differentially expressed genes
TCGA: The Cancer Genome Atlas
CXCL8: Chemokine (C-X-C motif) ligand 8
PPI: Protein–protein interaction
DGIdb: Drug-gene interaction database
DAVID: Database for annotation, visualization and integrated discovery
GO: Enrichment analyses of gene ontology
KEGG: Kyoto encyclopedia of genes and genomes
MCODE: Molecular complex detection
GEPIA: Gene expression profiling interactive analysis
COAD: Colon adenocarcinoma
READ: Rectal adenocarcinoma

## References

[1] Bray F, Ferlay J, Soerjomataram I, Siegel RL, Torre LA, Jemal A (2018). Global cancer statistics 2018: GLOBOCAN estimates of incidence and mortality worldwide for 36 cancers in 185 countries. CA Cancer J Clin.

[2] Marcuello M, Vymetalkova V, Neves RPL, Duran-Sanchon S, Vedeld HM, Tham E (2019). Circulating biomarkers for early detection and clinical management of colorectal cancer. Mol Aspects Med.

[3] Chen EX, Jonker DJ, Loree JM, Kennecke HF, Berry SR, Couture F et al. Effect of Combined immune checkpoint inhibition vs. best supportive care alone in patients with advanced colorectal cancer: The Canadian Cancer Trials Group CO.26 Study. JAMA Oncol 2020, 6(6):831–838. 10.1001/jamaoncol.2020.0910 PMC7206536 32379280

[4] Kim RD, Azad NS, Morse MA, Poplin E, Mahipal A, Tan B (2020). Phase II study of ensituximab, a novel chimeric monoclonal antibody, in adults with unresectable. Metastatic Colorectal Cancer Clin Cancer Res.

[5] Lafitte M, Sirvent A, Roche S (2020). Collagen kinase receptors as potential therapeutic targets in metastatic colon cancer. Front Oncol.

[6] Ibanez-Sanz G, Diez-Villanueva A, Alonso MH, Rodriguez-Moranta F, Perez-Gomez B, Bustamante M (2017). Risk model for colorectal cancer in spanish population using environmental and genetic factors: results from the MCC-Spain study. Sci Rep.

[7] Manzat-Saplacan RM, Mircea PA, Balacescu L, Chira RI, Berindan-Neagoe I, Balacescu O (2015). Can we change our microbiome to prevent colorectal cancer development?. Acta Oncol.

[8] Kosumi K, Hamada T, Koh H, Borowsky J, Bullman S, Twombly TS (2018). The amount of bifidobacterium genus in colorectal carcinoma tissue in relation to tumor characteristics and clinical outcome. Am J Pathol.

[9] Li SC, Lin HP, Chang JS, Shih CK. Lactobacillus acidophilus-Fermented Germinated Brown Rice Suppresses Preneoplastic Lesions of the Colon in Rats. Nutrients 2019, 11(11). 10.3390/nu11112718 PMC6893647 31717536

[10] Sobhani I, Tap J, Roudot-Thoraval F, Roperch JP, Letulle S, Langella P (2011). Microbial dysbiosis in colorectal cancer (CRC) patients. PLoS ONE.

[11] Geravand M, Fallah P, Yaghoobi MH, Soleimanifar F, Farid M, Zinatizadeh N (2019). Investigation of enterococcus faecalis population in patients with polyp and colorectal cancer in comparison of healthy individuals. Arq Gastroenterol.

[12] Lin C, Cai X, Zhang J, Wang W, Sheng Q, Hua H (2019). Role of gut microbiota in the development and treatment of colorectal cancer. Digestion.

[13] Song M, Chan AT, Sun J (2020). Influence of the gut microbiome, diet, and environment on risk of colorectal cancer. Gastroenterology.

[14] Si H, Yang Q, Hu H, Ding C, Wang H, Lin X. Colorectal cancer occurrence and treatment based on changes in intestinal flora. Semin Cancer Biol 2020. 10.1016/j.semcancer.2020.05.004 32404293

[15] Song X, Gao H, Lin Y, Yao Y, Zhu S, Wang J (2014). Alterations in the microbiota drive interleukin-17C production from intestinal epithelial cells to promote tumorigenesis. Immunity.

[16] Ajouz H, Mukherji D, Shamseddine A (2014). Secondary bile acids: an underrecognized cause of colon cancer. World J Surg Oncol.

[17] Liu H, Hunter L, Keselj V, Verspoor K (2013). Approximate subgraph matching-based literature mining for biomedical events and relations. PLoS ONE.

[18] Verspoor KM, Heo GE, Kang KY, Song M (2016). Establishing a baseline for literature mining human genetic variants and their relationships to disease cohorts. BMC Med Inform Decis Mak.

[19] Kveler K, Starosvetsky E, Ziv-Kenet A, Kalugny Y, Gorelik Y, Shalev-Malul G (2018). Immune-centric network of cytokines and cells in disease context identified by computational mining of PubMed. Nat Biotechnol.

[20] Tang Z, Li C, Kang B, Gao G, Li C, Zhang Z (2017). GEPIA: a web server for cancer and normal gene expression profiling and interactive analyses. Nucleic Acids Res.

[21] Tilg H, Adolph TE, Gerner RR, Moschen AR (2018). The intestinal microbiota in colorectal cancer. Cancer Cell.

[22] Sina C, Kemper C, Derer S (2018). The intestinal complement system in inflammatory bowel disease: Shaping intestinal barrier function. Semin Immunol.

[23] Wu J, Li Q, Fu X (2019). Fusobacterium nucleatum contributes to the carcinogenesis of colorectal cancer by inducing inflammation and suppressing host immunity. Transl Oncol.

[24] Zhang Y, Wang XL, Zhou M, Kang C, Lang HD, Chen MT (2018). Crosstalk between gut microbiota and Sirtuin-3 in colonic inflammation and tumorigenesis. Exp Mol Med.

[25] Cremonesi E, Governa V, Garzon JFG, Mele V, Amicarella F, Muraro MG (2018). Gut microbiota modulate T cell trafficking into human colorectal cancer. Gut.

[26] Zhang S, Yang Y, Weng W, Guo B, Cai G, Ma Y (2019). Fusobacterium nucleatum promotes chemoresistance to 5-fluorouracil by upregulation of BIRC3 expression in colorectal cancer. J Exp Clin Cancer Res.

[27] Xu Z, Zhang Y, Xu M, Zheng X, Lin M, Pan J (2019). Demethylation and overexpression of CSF2 are involved in immune response, chemotherapy resistance, and poor prognosis in colorectal cancer. Onco Targets Ther.

[28] Lee YY, Wu WJ, Huang CN, Li CC, Li WM, Yeh BW (2016). CSF2 Overexpression is associated with STAT5 phosphorylation and poor prognosis in patients with urothelial carcinoma. J Cancer.

[29] Du J, He Y, Li P, Wu W, Chen Y, Ruan H (2018). IL-8 regulates the doxorubicin resistance of colorectal cancer cells via modulation of multidrug resistance 1 (MDR1). Cancer Chemother Pharmacol.

[30] Cheng XS, Li YF, Tan J, Sun B, Xiao YC, Fang XB (2014). CCL20 and CXCL8 synergize to promote progression and poor survival outcome in patients with colorectal cancer by collaborative induction of the epithelial-mesenchymal transition. Cancer Lett.

[31] Alfaro C, Sanmamed MF, Rodriguez-Ruiz ME, Teijeira A, Onate C, Gonzalez A (2017). Interleukin-8 in cancer pathogenesis, treatment and follow-up. Cancer Treat Rev.

[32] Oladipo O, Conlon S, O'Grady A, Purcell C, Wilson C, Maxwell PJ (2011). The expression and prognostic impact of CXC-chemokines in stage II and III colorectal cancer epithelial and stromal tissue. Br J Cancer.

[33] Janjigian YY, Smit EF, Groen HJ, Horn L, Gettinger S, Camidge DR (2014). Dual inhibition of EGFR with afatinib and cetuximab in kinase inhibitor-resistant EGFR-mutant lung cancer with and without T790M mutations. Cancer Discov.

[34] Paul T, Schumann C, Rudiger S, Boeck S, Heinemann V, Kachele V (2014). Cytokine regulation by epidermal growth factor receptor inhibitors and epidermal growth factor receptor inhibitor associated skin toxicity in cancer patients. Eur J Cancer.

[35] Harrison SC, Smith AJ, Jones GT, Swerdlow DI, Rampuri R, Bown MJ (2013). Interleukin-6 receptor pathways in abdominal aortic aneurysm. Eur Heart J.

[36] Tseng-Rogenski SS, Hamaya Y, Choi DY, Carethers JM (2015). Interleukin 6 alters localization of hMSH3, leading to DNA mismatch repair defects in colorectal cancer cells. Gastroenterology.

[37] Rokavec M, Oner MG, Li H, Jackstadt R, Jiang L, Lodygin D (2014). IL-6R/STAT3/miR-34a feedback loop promotes EMT-mediated colorectal cancer invasion and metastasis. J Clin Invest.

[38] Moon SU, Kang MH, Sung JH, Kim JW, Lee JO, Kim YJ (2015). Effect of Smad3/4 on chemotherapeutic drug sensitivity in colorectal cancer cells. Oncol Rep.

[39] Wang J, Zhou J, Jiang C, Zheng J, Namba H, Chi P (2019). LNRRIL6, a novel long noncoding RNA, protects colorectal cancer cells by activating the IL-6-STAT3 pathway. Mol Oncol.

[40] Liu M, Guo S, Stiles JK (2011). The emerging role of CXCL10 in cancer (Review). Oncol Lett.

[41] Toiyama Y, Fujikawa H, Kawamura M, Matsushita K, Saigusa S, Tanaka K (2012). Evaluation of CXCL10 as a novel serum marker for predicting liver metastasis and prognosis in colorectal cancer. Int J Oncol.

